# Quantifying structural properties of forearm flexor muscles in individuals with hemiparetic cerebral palsy using diffusion tensor imaging

**DOI:** 10.14814/phy2.70404

**Published:** 2025-06-06

**Authors:** Divya Joshi, Alexandra Hruby, Julius P. A. Dewald, Carson Ingo

**Affiliations:** ^1^ Department of Biomedical Engineering Northwestern University Evanston Illinois USA; ^2^ Department of Physical Therapy and Human Movement Sciences Northwestern University Feinberg School of Medicine Chicago Illinois USA; ^3^ Department of Physical Medicine and Rehabilitation Northwestern University Feinberg School of Medicine Chicago Illinois USA; ^4^ Department of Neurology Northwestern University Feinberg School of Medicine Chicago Illinois USA

**Keywords:** cerebral palsy, diffusion tensor imaging, diffusivity metrics, muscle architecture, skeletal muscle

## Abstract

This study investigated diffusion tensor imaging (DTI) derived macro‐ and micro‐structural musculoskeletal adaptations in forearm flexor muscles in individuals with hemiparetic cerebral palsy (HCP) and typically developing (TD) individuals, and their relationship to reduced grip strength. In 14 individuals with HCP and 16 TD individuals, T1‐weighted and diffusion‐weighted magnetic resonance images of both forearms were acquired, and maximum grip strength was measured. In two forearm flexors, muscle volume, DTI‐based diffusivity metrics, and probabilistic tractography derived fascicle architecture was estimated. Linear mixed‐effects models evaluated interlimb differences in structural parameters and their impact on grip strength. In the HCP group, paretic muscles showed significant reductions in volume, diffusivity values, fascicle lengths, and physiological cross‐sectional area as compared to nonparetic forearm and TD participants. Furthermore, reduced muscle volume and diffusivity together explained 62% of the grip strength deficit. These findings demonstrate that decreased muscle volume and altered microstructure, as indicated by reduced diffusivity, contribute significantly to functional impairments in HCP. DTI‐based diffusivity metrics non‐invasively reveal crucial insights into pathophysiological changes in muscle tissue, such as muscle atrophy and fibrosis. Future therapies should focus on both muscle macro‐ and micro‐structural adaptations as targets to improve motor function in HCP.

## Graphical abstract

Hemiparetic cerebral palsy (HCP) results in micro‐ and macro‐structural adaptations in affected forearm muscles, which can be captured by diffusion tensor imaging (DTI) techniques and are related to reduced grip strength. These findings provide the foundation for DTI‐based, non‐invasive biomarkers of musculoskeletal changes following HCP and have important clinical implications.

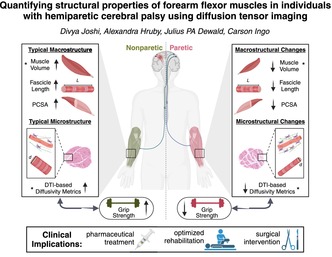

## 1 INTRODUCTION

Cerebral palsy (CP) is the most common movement disorder in childhood, caused by a brain injury that occurs around birth (Graham & Selber, 2003; Hanna et al., 2009). The hemiparetic cerebral palsy (HCP) subtype, which occurs in about 40% of all cases (Olivieri et al., 2013), results in significant reorganization in motor neural pathways (Eyre et al., 2001) and motor impairments on one side of the body, with severe weakness at distal joints such as the wrist and fingers (Sukal‐Moulton et al., 2014). The initial non‐progressive brain injury leads to secondary, progressive structural changes in the affected muscles, such as decreased muscle size (Barber et al., 2011; Handsfield et al., 2016; Malaiya et al., 2007; Noble, Fry, et al., 2014), altered muscle architecture (Lieber et al., 2017), and extracellular matrix (ECM) proliferation (Booth et al., 2001; Fridén & Lieber, 2003; Smith et al., 2011). Altered musculoskeletal properties in forearm muscles contribute to debilitating limitations in the functional use of the affected hand, but these underlying structural changes are not yet well understood (Mathewson & Lieber, 2015). Although the *in vivo* structure of leg muscles affected by CP has been studied more extensively (D'Souza et al., 2019; Handsfield et al., 2022; Sahrmann et al., 2019), there is little understanding of *in vivo* forearm muscle adaptations to inform neurorehabilitation treatment strategies for improved hand function in CP.

Intrinsic changes in macro‐ and micro‐structural properties of affected muscles are thought to contribute to weakness and decreased limb use (Hanssen et al., 2021). The most likely explanations are changes in muscle fascicle architecture, changes in the content and structure of ECM, or both (Mathewson & Lieber, 2015). A non‐invasive technique to efficiently characterize these properties *in vivo* is diffusion‐weighted MR‐based diffusion tensor imaging (DTI), which can overcome limitations posed by more traditional methods such as ultrasound, which is time‐consuming and limited in field‐of‐view for deeper structures, or histological analysis, which requires invasive biopsies (Bolsterlee et al., 2019; Damon et al., 2017; Oudeman et al., 2016). Our recent work established that probabilistic DTI tractography can capture valid and robust measurements of fascicle architecture in arm muscles (Joshi et al., 2024). Moreover, the use of DTI enables examination of diffusivity metrics, which illustrate the diffusion profile (restriction or lack thereof of water molecule diffusion) between intra‐ and extracellular compartments of muscle fibers (Berry et al., 2025). Previous work has shown that diffusion tensor scalars are sensitive to different pathologies in skeletal muscle tissue, including fibrosis or ECM proliferation (Berry et al., 2018), fiber atrophy (Berry et al., 2018; Cameron et al., 2023; Weedall et al., 2023; Yoon et al., 2018), and fatty infiltration (Hooijmans et al., 2015; Williams et al., 2013).

The overall goal of this study was to (1) quantify the interlimb differences in musculoskeletal structure in HCP and typically developing (TD) individuals, and (2) investigate how these interlimb differences are related to deficits in hand function. To achieve this goal, we implemented DTI and probabilistic tractography techniques (Joshi et al., 2024) in the forearm flexor muscles in the paretic (P) and nonparetic (NP) arms in individuals with HCP, as well as the dominant (D) and nondominant (ND) arms in TD controls. Because CP often causes an abnormally flexed hand posture, we focused on flexor muscles to better understand resulting changes in muscle structural morphology. Specifically, we analyzed the flexor carpi radialis (FCR), a wrist flexor, and the flexor digitorum profundus (FDP), a finger flexor, which both play significant roles in hand function and strength (Lieber & Fridén, 2000). We compared structural findings with maximal grip strength as a gross measure of functional ability of the flexor muscles. To the best of our knowledge, this is the first study to examine diffusivity metrics and tractography‐derived fascicle architecture estimates in arm muscles affected by CP.

## 2 METHODS

### 2.1 Participants

The study cohort included 30 individuals (18 females, 17.92 ± 9.0 years), consisting of 14 individuals with early onset (Sukal‐Moulton et al., 2013) HCP (7 females, 18.67 ± 10.2 years) and 16 TD individuals (11 females, 17.27 ± 8.1 years). All individuals were between 6 and 40 years old and had motor impairment confined to one side (HCP group) or no detectable motor impairment (TD group). We excluded individuals who: (1) had a history of botulinum toxin injections to any muscles of the upper limb within 6 months of testing, (2) had a history of surgery in the upper extremity within 1 year of testing, (3) had significant concurrent medical problems (including epilepsy or seizures), and/or (4) used medications known to suppress central nervous system activity. Though nonsteroidal anti‐inflammatory drugs (NSAIDs) or other pain‐relieving medications were not included as an official exclusion criterion, data collection was immediately stopped if participants expressed feeling pain. All participants and/or their guardians provided informed, written consent (and assent, for minors) to participate in this study, which was approved by the institutional review board of Northwestern University.

### 2.2 MR image acquisition and preprocessing

**FIGURE 1:**
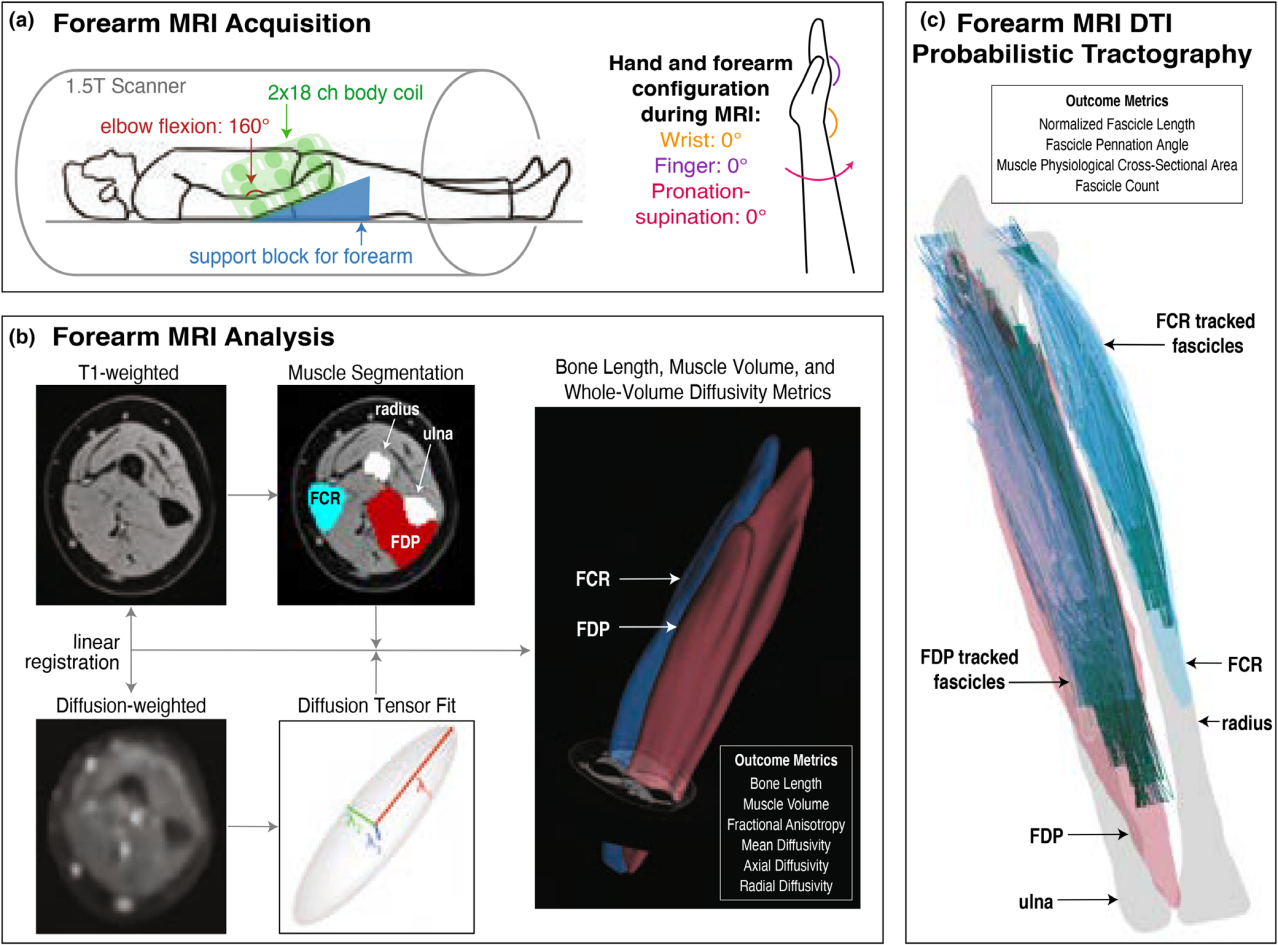
Pipeline of MR image data acquisition and analysis. (a) Participant positioning in 1.5 T MR scanner with 2 × 18 channel body coil around the forearm, with the elbow at 160° flexion and the hand and fingers placed in an MRI‐safe orthosis to hold the wrist at 0°, fingers at 0°, and forearm pronation‐supination at 0°. (b) MR data preprocessing, including segmentation of regions of interest from T1 images and diffusion tensor estimation from dMR volumes to calculate bone length, muscle volume, and whole‐volume diffusivity metrics. (c) Example of muscle fascicles reconstructed from valid tracts, shown with 3D bone and muscle surfaces, used to calculate fascicle architecture metrics.

In all participants, T1‐weighted 3D VIBE (TR = 16 ms, TE = 7.16 ms, FOV = 256 × 304 mm^2^, voxel = 0.78 × 0.78 × 3 mm^3^) and diffusion‐weighted (DW) spin‐echo EPI (TR = 8500 ms, TE = 48 ms, FOV = 250 × 250 mm^2^, voxel = 1.25 × 1.25 × 6.5 mm^3^, 12 b = 400 s/mm^2^ directions with 3 averages each, 10 b = 0 s/mm^2^ volumes) images were acquired of both forearms on a 1.5 T scanner (Joshi et al., 2024) (Figure 1a). DW image preprocessing was carried out in FSL (v6.0, https://fsl.fmrib.ox.ac.uk/fsl/fslwiki) (Smith et al., 2004) according to previously established methods (Joshi et al., 2024), including denoising (Aja‐Fernández et al., 2008), affine registration (Andersson et al., 2016; Andersson & Sotiropoulos, 2016), correction for diffusion gradient directions (Andersson et al., 2016; Andersson & Sotiropoulos, 2016), registration between T1 and DW image space (Jenkinson et al., 2002; Jenkinson & Smith, 2001), and diffusion tensor fitting (Behrens et al., 2003).

### 2.3 MR image analysis

#### 2.3.1 Identification of regions of interest

The outer boundaries and inner volumes of the FCR and the FDP were segmented in T1 space and registered to diffusion image space (Figure 1b), from which muscle volumes were measured. The Euclidean distance between the proximal and distal points of the ulna and radius was calculated as the bone length. Finally, the lateral edge and aponeurosis of each muscle of interest were identified according to previously published methods (Joshi et al., 2024).

#### 2.3.2 Diffusivity metric measurement

For each muscle, whole‐volume fractional anisotropy (FA), mean diffusivity (MD), axial diffusivity (AD), and radial diffusivity (RD) were estimated by calculating the metric within each voxel from its fitted diffusion tensor and averaged across all voxels in the muscle volume (Figure 1b). By definition, whole‐volume diffusivity metrics are scaled to a uniform voxel size (Soares et al., 2013), so further normalization to muscle volume is not required.

#### 2.3.3 Fascicle architecture measurement

For each muscle, probabilistic tractography was performed using FSL's PROBTRACKX (Behrens et al., 2003, 2007) in the FCR and FDP muscles using optimal parameters and methods established previously (Joshi et al., 2024). Tracts were seeded from each voxel in the muscle volume region, propagated bidirectionally, terminated when exiting the muscle boundary, and must have made contact with the muscle boundary. Probabilistic tractography parameters included a minimum FA threshold of 0, a step size of 0.5 mm, and a turning angle of 90° (Joshi et al., 2024). Tracts were filtered according to predetermined anatomical constraints (Joshi et al., 2024), and remaining valid tracts represented muscle fascicle reconstructions. From this point on, the term fascicle refers to a valid tract reconstruction. From these reconstructions, fascicle count, lengths, and pennation angles were quantified (Joshi et al., 2024) (Figure 1c). Physiological cross‐sectional area (PCSA) was calculated according to Equation (1)

(1) $$\text{PCSA}=\frac{\text{Muscle Volume}*\cos\left(\mu_{\text{Pennation Angle}}\right)}{\mu_{\text{Fascicle Length}}},$$

where *μ* refers to the average value across all valid muscle fascicles (Damon et al., 2017). Fascicle lengths were normalized to the radius (FCR) or ulna (FDP) length to account for possible differences in limb size between arms. Examples of 3D fascicle reconstructions are shown in Figure S1. We would like to note that Equation (1) is an adaptation of the canonical definition of PCSA, which necessitates knowledge of the optimal fiber length and thus sarcomere length when the muscle is at its optimal length (Rockenfeller et al., 2024). However, sarcomere lengths are not able to be measured non‐invasively using MR techniques. Therefore, we have modified this calculation to estimate PCSA as shown in Equation (1), using average fascicle pennation angles and lengths estimated from tractography methods, as is standard in DTI muscle studies (Damon et al., 2017; D'Souza et al., 2019; Sahrmann et al., 2019).

### 2.4 Functional measurement and clinical assessments

#### 2.4.1 Grip strength

In all participants, two handheld dynamometers were used to measure maximum grip strength for each hand (Hruby et al., 2023).

#### 2.4.2 Fugl‐Meyer Assessment

The Upper Extremity Fugl‐Meyer Assessment (FMA) (Fugl Meyer et al., 1975) was evaluated in all participants with HCP. Scores from Sections B and C were summed for a wrist and hand function score.

#### 2.4.3 Modified Ashworth Scale

The Modified Ashworth Scale (MAS) assessment (Ansari et al., 2008) was completed in all participants with HCP. The test was performed on the wrist to evaluate forearm flexor muscles' resistance to elongation.

#### 2.4.4 GMFCS and BFMF scores

All participants with HCP were retrospectively categorized into Gross Motor Function Classification System (GMFCS) (Palisano et al., 1997) and Bimanual Fine Motor Function (BFMF) (Elvrum et al., 2016) levels.

### 2.5 Statistical analyses

All statistical tests were performed in R version 4.2.2 (R Foundation for Statistical Computing, Vienna, Austria).

#### 2.5.1 Participant characteristics

Two sample *t*‐tests were performed to evaluate differences in age and grip strength between the HCP and TD groups. A chi‐squared test was performed to evaluate differences in sex between the HCP and TD groups.

#### 2.5.2 MR‐derived outcome measures

Outcome measures included musculoskeletal parameters (bone length, muscle volume), fascicle architecture estimates (normalized fascicle length, fascicle pennation angle, fascicle count, and PCSA), and diffusivity metrics (FA, MD, AD, and RD). For each outcome measure, values were averaged together across the FCR and FDP muscles, weighted by the volumetric contribution of each muscle. Linear mixed effects (LME) models were used for each outcome measure to determine whether the outcome measure was significantly different between arms according to Equation (2).
(2)

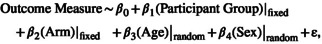

where each LME model consisted of the outcome measure as the dependent variable, participant group (HCP or TD) and arm (D/NP or ND/P) as fixed effects, and age and sex as random effects. Because the cohort was age‐ and sex‐balanced and the study focused on within‐subject comparisons, age and sex were modeled as random effects to account for between‐subject variability. Additionally, the sample size was not powered to detect between‐group differences by age or sex, so formal subgroup analyses were not performed.

#### 2.5.3 Correlation analysis

A percent deficit (PD) in each participant was found for each MR‐derived outcome measure according to Equation (3) (HCP group) or Equation (4) (TD group):

(3) $$\begin{array}{c} \text{Percent Deficit}\;{\left(\mathrm{PD}\right)}_{\text{Outcome Measure},\mathrm{HCP}}\\ =\frac{\mu_{\text{Outcome Measure},\mathrm{NP}}-\mu_{\text{Outcome Measure},P}}{\mu_{\text{Outcome Measure},\mathrm{NP}}}*100\%, \end{array}$$

(4) $$\begin{array}{c} \text{Percent Deficit}\;{\left(\mathrm{PD}\right)}_{\text{Outcome Measure},\mathrm{TD}}\\ =\frac{\mu_{\text{Outcome Measure},D}-\mu_{\text{Outcome Measure},\mathrm{ND}}}{\mu_{\text{Outcome Measure},D}}*100\%, \end{array}$$

where *μ*_Outcome Measure_ refers to the average value of each outcome measure in the corresponding arm (NP or P in the HCP group, and D or ND in the TD group). Pearson correlation coefficients were calculated between PD_Grip Strength_ and the PD for each outcome measure. Variables that were significantly correlated to PD_Grip Strength_ were chosen as fixed effects for an integrated LME model that included PD_Grip Strength_ as the dependent variable and age and sex as random effects.

For each statistical test, the significance level was set at *p* < 0.05.

### 2.6 Scientific rigor

This study was designed to maintain scientific rigor through careful methodological considerations. Randomization was not applicable, as group assignment was based on clinical diagnosis (HCP vs. TD). However, efforts were made to match groups on key demographic variables, including age and sex, to minimize confounding effects. All data analysis was completed in a blinded manner to ensure objective interpretation of results and reduce bias related to participant group assignment. To address attrition, participant retention strategies were employed, including follow‐up reminders and flexible scheduling. Note that of the 36 individuals recruited for this study, 6 were removed due to MR motion artifacts, unclear HCP diagnosis, or incomplete data collection. Due to the constraints of recruiting individuals with a specific diagnosis, a formal power analysis was not performed. Instead, the sample size was determined by the maximum number of eligible participants available during the study period. All code used in data analysis is available in the Supplemental Material.

## 3 RESULTS

Table 1 shows a summary of the study cohort, including age, sex, and clinical assessment scores, when applicable. 13 out of the 14 individuals with HCP had mild to moderate levels of impairment (BFMF: I‐IIa; MAS: 0–2; FMA hand: 9–24). One participant with HCP had severe impairment in the upper extremity (BFMF: IIIa; MAS: 4; FMA hand: 4). There was no significant difference in age and sex between the HCP and TD groups (*p* > 0.05). However, PD_Grip Strength_ was about five times significantly greater in the HCP group than in the TD group (*p* = 0.002).

**TABLE 1:** Summary of study participants.

Patient group	Age	Sex	GMFCS (if applicable)	BFMF (if applicable)	MAS (if applicable)	FMA wrist/hand (if applicable)
(A) Individual participant description						
HCP	10 y 2 m	F	I	I	1+	19
HCP	11 y 6 m	M	I	IIa	2	9
HCP	13 y 7 m	F	I	I	0	24
HCP	24 y 10 m	M	II	IIa	2	11
HCP	16 y 9 m	M	I	IIa	2	13
HCP	20 y 1 m	F	I	I	1	24
HCP	10 y 9 m	M	I	I	0	21
HCP	8 y 1 m	F	I	I	0	24
HCP	12 y 9 m	F	II	IIIa	4	4
HCP	13 y 1 m	M	I	I	0	24
HCP	39 y 2 m	M	I	I	0	22
HCP	18 y 3 m	F	I	I	0	24
HCP	24 y 6 m	F	I	I	1	23
HCP	39 y 8 m	M	I	I	1+	24
TD	31 y 1 m	F				
TD	10 y 3 m	F				
TD	20 y 7 m	M				
TD	6 y 6 m	F				
TD	11 y 8 m	M				
TD	24 y 3 m	F				
TD	10 y 3 m	F				
TD	12 y 2 m	M				
TD	11 y 1 m	F				
TD	28 y 2 m	F				
TD	31 y 2 m	M				
TD	21 y 9 m	M				
TD	15 y 5 m	F				
TD	20 y 2 m	F				
TD	12 y 5 m	F				
TD	9 y 4 m	F				

	HCP	TD	Total
(B) Study Cohort Summary			
*n*	14	16	30
Age (*p* > 0.05 between HCP and TD)	18.67 ± 10.2 y	17.27 ± 8.1 y	17.92 ± 9.0 y
Sex (*p* > 0.05 between HCP and TD)	7F, 7M	11F, 5M	18F, 12M
Grip Strength Percent Deficit (*p* = 0.00023 between HCP and TD)	48.63% ± 21.44%	9.87% ± 26.5%	27.87% ± 30.9%

*Note*: (A) Participant‐level demographic details. In participants with HCP, Gross Motor Classification System (GMFCS), Bimanual Fine Motor Function (BFMF), Modified Ashworth Scale (MAS), and Fugl‐Meyer Assessment (FMA) wrist and hand subsection scores are shown. Note that the maximum FMA wrist/hand subsection score is 24. (B) Summary of the entire study cohort, including the number of participants (*n*), age, sex, and grip strength percent deficit in each group and the total cohort. For age, sex, and grip strength deficit, *p*‐values for t‐tests (age and grip strength deficit) or chi‐squared tests (sex) between the HCP and TD groups are shown.

**FIGURE 2:**
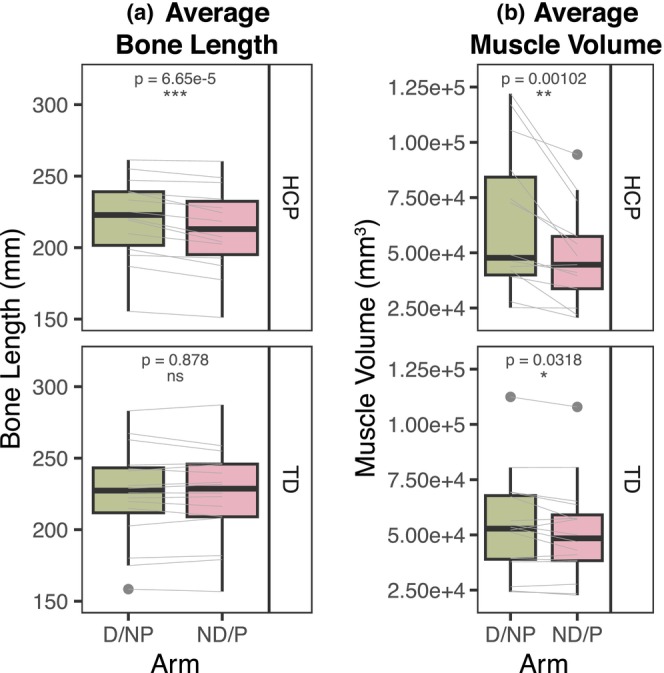
Anthropometric measures derived from T1‐weighted image segmentations. **(a)** Bone length (mm), averaged between the ulna and radius bone lengths. **(b)** Muscle volume (mm^3^), averaged between the FCR and FDP muscle volumes. **(a, b)** *Top:* HCP group, *bottom:* TD group, green: TD dominant (D) or HCP non‐paretic (NP) arm, pink: TD nondominant (ND) or HCP paretic (P) arm. Black boxplots represent distribution of group data, while gray lines represent individual participant data paired between arms. P‐values shown represent significance of interlimb difference determined from linear mixed effects models. ns, no significant difference; *p < 0.05, **p < 0.01, and ***p < 0.001.

Figure 2 shows macrostructural musculoskeletal parameters in both groups. In the HCP group, the paretic limb bone length was 3.4% shorter than that of the nonparetic limb (*p* = 6.65e‐5), while there was no interlimb difference in the TD group (*p* = 0.878) (Figure 2a). There was a significant reduction in muscle volume in both the HCP paretic limb and the TD nondominant limb, as compared to the nonparetic/dominant limb (*p* = 0.00102 and *p* = 0.0318, respectively), although there was a greater mean decrease in the HCP group than in the TD group (27.0% and 4.5%, respectively) (Figure 2b).

**FIGURE 3:**
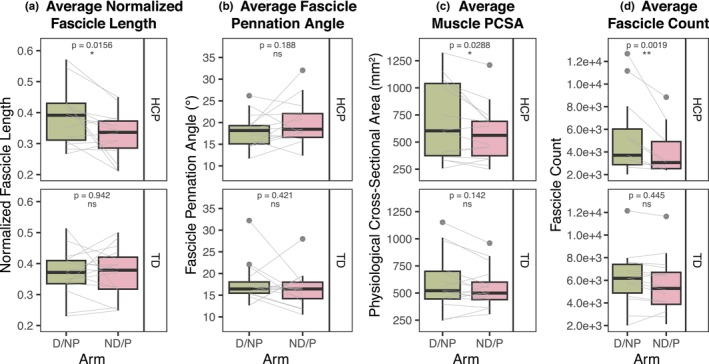
Probabilistic tractography derived measures. (a) Fascicle lengths normalized to bone length, (b) fascicle pennation angles (°), (c) physiological cross‐sectional area (PCSA) (mm^2^), and (d) fascicle count, averaged between the FCR and FDP muscles. (a–d) Top: HCP group, bottom: TD group, green: TD dominant (D) or HCP non‐paretic (NP) arm, pink: TD nondominant (ND) or HCP paretic (P) arm. Black boxplots represent distribution of group data, while gray lines represent individual participant data paired between arms. *p*‐values shown represent significance of interlimb difference determined from linear mixed effects models. ns, no significant difference, *p < 0.05; **p < 0.01; ***p < 0.001.

Forearm flexor fascicle architecture parameters derived from tractography results are shown in Figure 3. In the HCP group, the paretic arm showed a 15.8% decrease in normalized fascicle lengths (*p* = 0.0156) (Figure 3a), no change in fascicle pennation angles (*p* = 0.188) (Figure 3b), and a 15.9% decrease in PCSA (*p* = 0.0288) (Figure 3c). Fascicle count was decreased by 34.1% in the HCP group (*p* = 0.00192) (Figure 3d) but showed no significant interlimb difference when normalized by muscle volume. There were no significant interlimb differences for any fascicle architecture parameters in the TD group.

**FIGURE 4:**
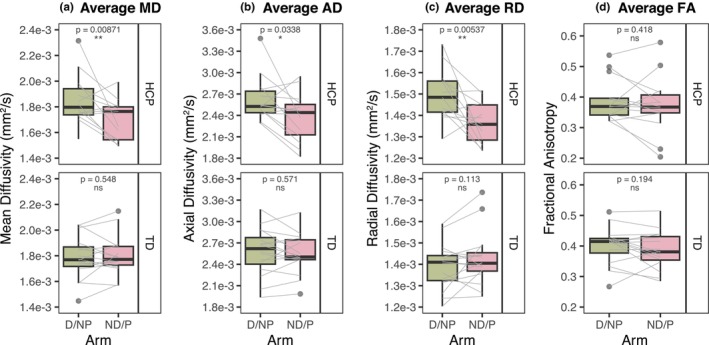
DTI derived diffusivity metrics. (a) Mean diffusivity (MD) (mm^2^/s), (b) axial diffusivity (AD) (mm^2^/s), (c) radial diffusivity (RD) (mm^2^/s), and (d) fractional anisotropy (FA), averaged between the FCR and FDP muscles. (a–d) Top: HCP group, bottom: TD group, green: TD dominant (D) or HCP non‐paretic (NP) arm, pink: TD nondominant (ND) or HCP paretic (P) arm. Black boxplots represent distribution of group data, while gray lines represent individual participant data paired between arms. *p*‐values shown represent significance of interlimb difference determined from linear mixed effects models. ns, no significant difference; *p < 0.05, **p < 0.01; ***p < 0.001.

Figure 4 shows the diffusivity metrics in the forearm flexor muscles. In the HCP group, the paretic arm muscles had MD, AD, and RD values that were all decreased by about 9.3% from the nonparetic arm (*p* = 0.00871, *p* = 0.0338, and *p* = 0.00537, respectively) (Figure 4a–c), while FA showed no interlimb differences (*p* = 0.418) (Figure 4d). For the TD group, there were no significant interlimb differences in MD, AD, RD, or FA (*p* > 0.05 for all) (Figure 4a–d).

**FIGURE 5:**
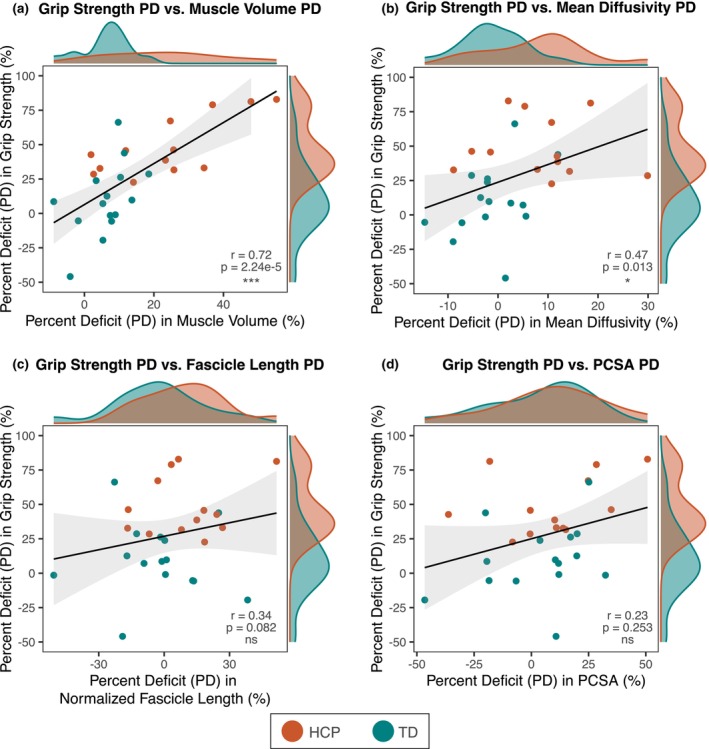
Relationships between percent deficits (PD) (%) in grip strength and muscle macro‐structural and micro‐structural measures. (a) Grip strength PD versus muscle volume PD, (b) grip strength PD versus mean diffusivity (MD) PD, (c) grip strength PD versus normalized fascicle length PD, and (d) grip strength PD versus physiological cross‐sectional area (PCSA) PD. (a–d): Red dots represent individuals with HCP and green dots represent TD individuals. Distributions of x‐ and y‐data, by group, are shown at the top and right of each graph, respectively. Linear regression lines fitted to the data are shown in black, with 95% confidence intervals shown in light gray. Pearson correlation coefficients (*r*) and associated *p*‐values are shown for each comparison. ns, no significant difference; *p < 0.05; **p < 0.01; ***p < 0.001.

The relationships between PD_Grip Strength_ and PD_Muscle Volume_, PD_MD_, PD_Normalized Fascicle Length_, and PD_PCSA_ are shown in Figure 5. PD_Grip Strength_ is significantly correlated with PD_Muscle Volume_ (*r* = 0.72, *p* = 2.24e‐5) (Figure 5a) and PD_MD_ (*r* = 0.47, *p* = 0.013) (Figure 5b), but is not correlated with PD_Normalized Fascicle Length_ (*r* = 0.34, *p* = 0.082) (Figure 5c) or PD_PCSA_ (*r* = 0.23, *p* = 0.254) (Figure 5d). Furthermore, PD_MD_ is not significantly correlated with PD_Muscle Volume_ (*r* = 0.32, *p* = 0.1). PD_AD_ and PD_RD_ showed similar results as PD_MD_, so only PD_MD_ is shown. The PDs in all other outcome measures were not significantly correlated to PD_Grip Strength_. Because PD_Muscle Volume_ and PD_MD_ were each significantly correlated with PD_Grip Strength_ in the separate correlation analyses, they were both used as fixed effects in an integrated LME model according to Equation (5).
(5)

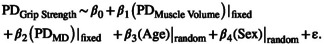




The integrated LME model showed that both PD_Muscle Volume_ and PD_MD_ significantly contribute to PD_Grip Strength_ (PD_Muscle Volume_: *β*_1_ = 1.27, *p* = 3.45e‐5; PD_MD_: *β*_2_ = 0.843, *p* = 0.0472; *r* ^2^ = 0.62). For comparison, when investigating models with only one predictor for PD_Grip Strength_, the LME model with PD_Muscle Volume_ as the only fixed effect had an *r* ^2^ value of 0.52, while the LME model with PD_MD_ as the only fixed effect had an *r* ^2^ value of 0.22.

## 4 DISCUSSION

The current study investigated macro‐ and micro‐structural parameters, along with DTI‐based diffusivity metrics, in forearm flexor muscles of individuals with HCP compared to TD controls. To investigate the effect these had on function, interlimb differences in muscle structural parameters were compared to the interlimb difference in maximum grip strength. We found that macro‐structural measures (bone length, muscle volume, fascicle length, and PCSA) and diffusivity metrics, indicative of micro‐structural parameters, were significantly reduced in the paretic forearm muscles in the HCP group, but not in the TD group. Furthermore, deficits in structural parameters were significantly correlated with and contributed to observed deficits in grip strength. Here, we discuss the implications of our findings and their relevance to clinical practice and future research.

Individuals with HCP exhibited substantial reductions in bone length in their paretic limbs compared to their nonparetic limbs, which were not observed in the TD group. On average, the paretic limb's ulna and radius bones were shorter by about 7.5 mm, in line with previous reports in CP (Kim & Son, 2022; Riad et al., 2010). Additionally, though there was a significant interlimb difference in muscle volume in both groups, the percent decrease was about six times higher in HCP than in TD. In HCP, our finding that the paretic forearm flexors have a 27% smaller muscle volume than the nonparetic limb is in line with similar findings of a 20%–28% volume deficit in affected calf flexor muscles (Barber et al., 2011; Handsfield et al., 2016; Malaiya et al., 2007; Noble, Fry, et al., 2014). The interlimb discrepancy in TD individuals is likely due to the greater use of the dominant wrist and fingers, resulting in slightly larger flexor muscle volume (Abe & Loenneke, 2015; Sanchis‐Moysi et al., 2012). These results further establish that the initial brain insult in CP has negative downstream effects on the musculoskeletal system's development, particularly in distal body parts (Demir et al., 2006; Kim & Son, 2022; Zonta et al., 2009).

Though smaller muscle volume has traditionally been thought of as the primary non‐neural cause of weakness in CP (Elder et al., 2003), Hanssen et al. recently showed that only 22%–57% of weakness at the knee and ankle can be explained by reduced muscle volume, with the remainder likely due to altered neural control and/or intrinsic muscle alterations (Hanssen et al., 2021). Specifically, observed muscle weakness may be explained in part by increased non‐contractile tissue within an already reduced muscle volume, resulting in a lower proportion of contractile tissue and thus greater weakness (Hanssen et al., 2021). Other studies have shown significant associations between the amount of non‐contractile muscle tissue and weakness in the lower limb (Baum et al., 2016; van den Noort et al., 2022), though the composition of this non‐contractile tissue is still unclear (Von Walden et al., 2017). Our findings of lower diffusivity in affected muscles may shed light on potential pathophysiologic changes in individuals with CP.

In the HCP group's paretic flexor muscles, MD, AD, and RD decreased by similar amounts, while FA remained largely unchanged, as compared to the nonparetic arm. These results in the paretic limb are indicative of increased hindrances that water molecules encounter in all directions, though the anisotropic degree of water diffusion stays the same. That is to say, the diffusion tensor remains in the same overall geometric shape, but all three tensor eigenvalues decrease in displacement. Furthermore, diffusivity metrics are already normalized to voxel size, suggesting that any alteration in diffusivity inherently indicates potential changes in tissue composition within each unit muscle volume. Decreased diffusivity in skeletal muscle may be indicative of fibrosis or excessive accumulation of ECM. Histologic studies have shown that collagen content, the main component of ECM, was three‐fold higher in affected forearm muscles of children with CP compared to TD children (De Bruin et al., 2014), and that increased collagen content is significantly correlated with motor impairment, as measured by MAS (Booth et al., 2001; Mathewson & Lieber, 2015). Collagen molecules are highly restrictive to diffusion (Kihara et al., 2013; Ramanujan et al., 2002), and their accumulation, along with increased extracellular space (Berry et al., 2018), would decrease overall diffusivity in muscle tissue.

Along with ECM proliferation, fiber atrophy—or a reduction in fiber diameter—has been observed in muscles affected by CP (Mathewson & Lieber, 2015; Tosi et al., 2009). Previous studies found that fiber atrophy was associated with increased FA and decreased MD (Cameron et al., 2023; Weedall et al., 2023; Yoon et al., 2018), likely explained by reduced intracellular space caused by smaller fiber diameters (Berry et al., 2025). Another histological study on rat muscle specimens showed that increased FA along with decreased MD and RD were related to reduced fiber diameter and increased ECM volume. In particular, RD, the diffusivity in the secondary and tertiary directions, was positively correlated with fiber diameter (Yu et al., 2021). Therefore, our finding of decreased diffusivity may be indicative of fiber atrophy in the affected muscles, though we found no differences in FA. In this study, 12 diffusion gradient directions were employed in the MR sequence, as is standard in skeletal muscle DTI (Oudeman et al., 2016) to ensure reasonable scan time. However, it has been suggested that a higher number of gradient directions can result in more robust FA measures (Chianca et al., 2017), indicating that perhaps slight increases in FA were not captured with our current protocol of 12 directions.

Finally, decreased diffusivity may be due to fatty infiltration (Hooijmans et al., 2015; Williams et al., 2013). Individuals with CP have increased intramuscular fat in leg muscles compared to their TD peers, which increases with severity level (Kihara et al., 2013; Ramanujan et al., 2002). However, post‐hoc fat fraction analysis in participants with HCP and TD participants showed no meaningful interlimb increase in intramuscular fat (Figure S2), and the fat fraction range of 3%–5% observed in both arms is much lower than the previously established threshold for accurate diffusivity metric measurement, 45% (Williams et al., 2013). Additionally, diffusion‐weighted images in the current study were acquired with a SPectral Attenuated Inversion Recovery (SPAIR) sequence to suppress any contributions from fat to the diffusion signal. Therefore, it can be assumed that any contribution from intramuscular fat has minimal contributions to the observed decreased diffusivity, particularly considering our study cohort, which primarily has mild impairments.

**FIGURE 6:**
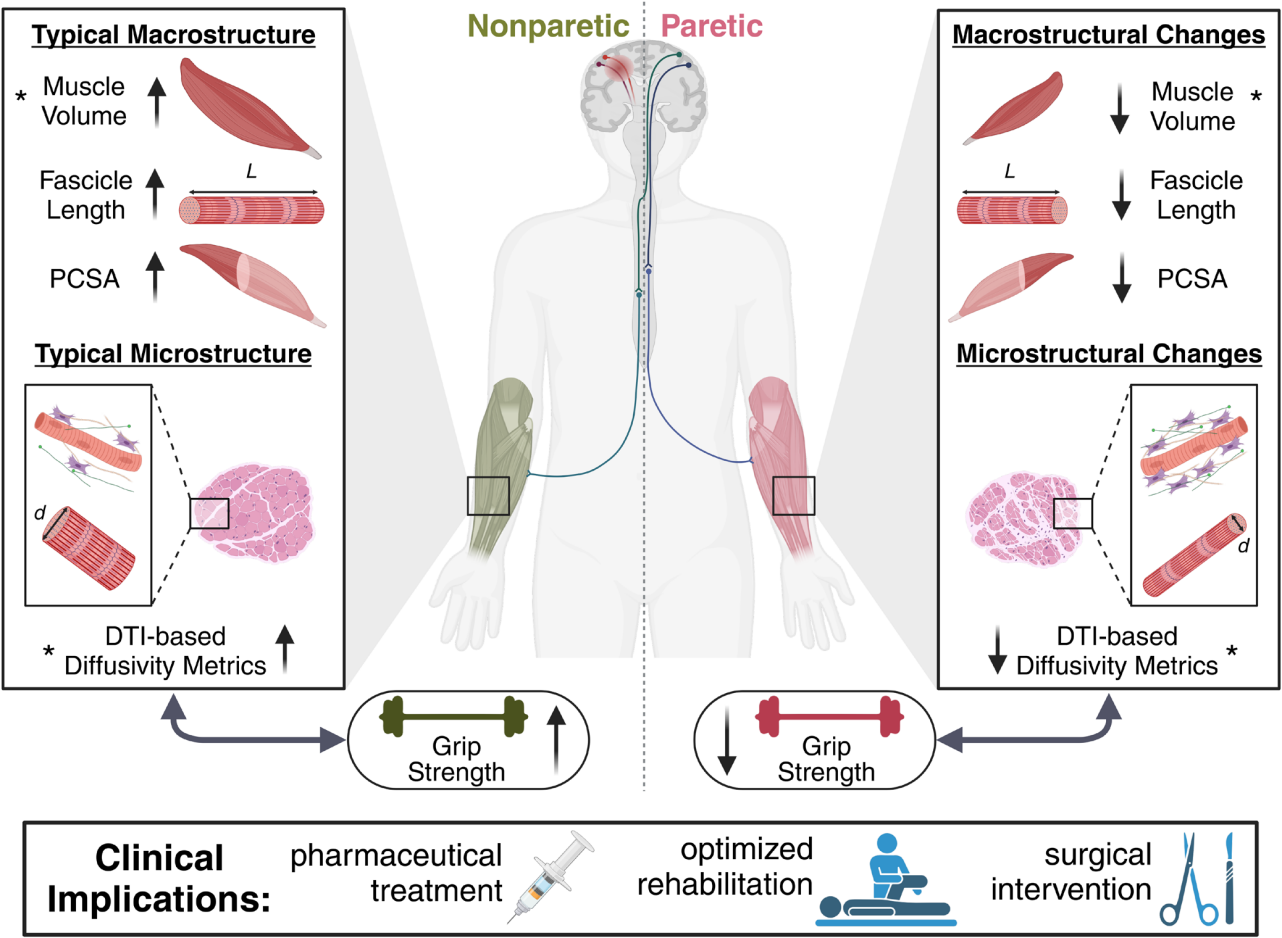
Schematic of adaptations in forearm musculoskeletal macrostructure and microstructure following hemiparetic cerebral palsy, and relationship to grip strength, a functional outcome. A *indicates parameters that showed significant correlations to grip strength. Possible clinical implications of this work are shown. Created using Biorender.com.

When investigating the relationship between muscle structural measures and grip strength, we found that muscle volume deficit explained 52%, in line with previous work (Hanssen et al., 2021), and MD deficit explained 22% of the variance in grip strength deficit. Moreover, both muscle volume deficit and MD deficit were significant predictors for grip strength deficit and, when modeled together, explained about 62% of the variance in grip strength deficit. Therefore, although reduced muscle volume plays a key role, non‐volume related intrinsic muscle adaptations also significantly affect observed weakness in HCP. Based on the above discussion, we postulate that muscles affected by HCP are likely to have greater ECM proliferation and decreased fiber diameter than their nonparetic counterparts. These pathophysiologic changes would exacerbate the already substantial weakness caused by reduced muscle mass (Figure 6).

In the current study, we found that the paretic forearm flexors had shorter fascicles but unchanged pennation angles, resulting in a reduced PCSA. Though previous reports of fascicle lengths and pennation angles in muscles affected by CP have yielded inconsistent results (Chianca et al., 2017; D'Souza et al., 2020; Handsfield et al., 2016; Mohagheghi et al., 2007, 2008; Noble, Charles‐Edwards, et al., 2014; Noble c, Fry, et al., 2014; Tosi et al., 2009; Yu et al., 2021), the current understanding is that PCSA is reduced following CP and contributes to weakness (Handsfield et al., 2022). However, the formal mathematical definition of PCSA includes sarcomere length, which cannot be estimated with MRI alone (Mathewson & Lieber, 2015), as is the case in this study and previous work (D'Souza et al., 2019; Sahrmann et al., 2019). Furthermore, in our calculation of PCSA, we used the mean fascicle length and pennation angle, as has been done previously (D'Souza et al., 2019; Sahrmann et al., 2019), but it has been previously shown that fascicle lengths and pennation angles may not follow a normal distribution across a muscle's volume (Joshi et al., 2024; Murray et al., 2000; Rockenfeller et al., 2024). To ensure that this phenomenon did not significantly affect the PCSA estimates, we recalculated PCSA using both the median and mode fascicle length and pennation angle in each participant and found no significant changes from the results shown here.

Nevertheless, if the shortened fascicles measured here also had abnormally lengthened sarcomeres, as has previously been observed in CP (Lieber & Fridén, 2002; Smith et al., 2011), then the optimal fascicle length (fascicle length normalized to sarcomere length) would be further reduced (Mathewson & Lieber, 2015). Therefore, the non‐significant relationship between deficits in PCSA and grip strength observed in this study is likely due to an underestimation of the interlimb difference in PCSA resulting from the necessary exclusion of sarcomere length data. Moreover, when grouping estimates from both arms together rather than focusing on the percent deficits, we found that PCSA was significantly correlated with grip strength (*r* = 0.54, *p* = 1.79e‐5) (Figure S3). This further indicates that the observed absence of a significant relationship between deficits in PCSA and grip strength likely stems from unaccounted differences in optimal sarcomere length between the paretic and nonparetic muscles in the HCP group.

Several limitations of the current study should be acknowledged. First, though DTI‐based diffusivity metrics can indirectly provide an idea of tissue‐level changes, they are highly sensitive but not specific. Therefore, without performing a comparative histology study, we cannot identify the specific pathology in the muscle tissue. However, given the difficulty of obtaining biopsy samples in a pediatric population, DTI presents an effective non‐invasive tool to understand the extent of muscle tissue adaptations in individuals with CP. Second, we relate MR results to grip strength, which is a gross measure of distal impairment and weakness. Future work should relate structural findings to high‐resolution measures of wrist and finger dexterity, as well as passive stiffness and range of motion at both joints. Third, though pain levels were not considered in this study, extreme pain may reduce function and induce muscle inflammation in the affected limb(s). Thus, future studies should consider regular use of NSAIDs or other pain‐relieving medications as a component of the exclusion criteria. Finally, this study aimed to non‐invasively study changes in contractile tissue following CP, such as increased ECM, so we primarily recruited individuals with mild impairments. This work can be expanded upon in the future to include participants with more heterogeneous levels of impairment, in which both ECM and fat infiltration may contribute to reduced function, to understand changes in more severely affected muscles. Additionally, a larger cohort would provide the statistical power necessary to more rigorously examine the effects of sex and age on interlimb differences in muscle structure and function.

In conclusion, our findings provide novel insights into the macro‐ and micro‐structural characteristics of forearm flexor muscles in individuals with HCP. Specifically, we found that reduced diffusivity in affected muscles may indicate a greater amount of non‐contractile tissue, contributing to weakness in HCP. A non‐invasive tool like DTI can be used to identify structural adaptations in muscle, which can then inform the development of targeted orthopedic, rehabilitation, and pharmacological interventions aimed at preserving muscle structure and thus optimizing motor function (Figure 6). First, knowledge of how musculoskeletal structural parameters are compromised on an individual basis can lead to more precise and personalized surgical management (Graham & Selber, 2003). Second, if the progression of structural musculoskeletal adaptations is better understood, targeted rehabilitation techniques, such as encouraging the use of paretic limbs at a young age, could be employed before the development of irreversible deficits such as the proliferation of ECM and contractures (Haak et al., 2009). Finally, if there is indeed a shortening of fascicles and proliferation of ECM in affected muscles—as suggested by the results presented here—drugs could be administered that stimulate serial addition of sarcomeres and/or break down collagen molecules, such as hyaluronidase injections (Raghavan et al., 2016). This study's findings regarding diffusivity metrics in skeletal muscles affected by CP can be expanded upon for a greater understanding of structural muscle tissue adaptations underlying functional impairment in CP.

## AUTHOR CONTRIBUTIONS

DJ, JPAD, and CI conceived and designed the research; DJ and AH performed experiments and analyzed data; DJ, JPAD, and CI interpreted the results of the experiments; DJ prepared figures; DJ drafted the manuscript; DJ, AH, JPAD, and CI edited and revised the manuscript; DJ, AH, JPAD, and CI approved the final version of themanuscript.

## FUNDING INFORMATION

This work was funded by National Institutes of Health grants R01NS126509, R03HD094615, R01NS058667, and F31HD110236.

## CONFLICT OF INTEREST STATEMENT

The authors have no conflicts to disclose.

## ETHICS STATEMENT

This study was approved by the Institutional Review Board of the Northwestern University (IRB STU00003762).

## Supporting information


Appendix S1.

## Data Availability

Source data for this study are not publicly available due to privacy or ethical restrictions. The source data are available to verified researchers upon request by contacting the corresponding author.
