# Supplementary material for: Quantifying structural properties of forearm flexor muscles in individuals with hemiparetic cerebral palsy using diffusion tensor imaging
Source: Physiol Rep. 2025 Jun 6;13(11):e70404. doi: 10.14814/phy2.70404 (PMC12141930; doi:10.14814/phy2.70404)
Supplement: Supplementary file 1 — Appendix S1. [file PHY2-13-e70404-s001.pdf]

Examples of Forearm Flexor Muscle Fiber Reconstructions

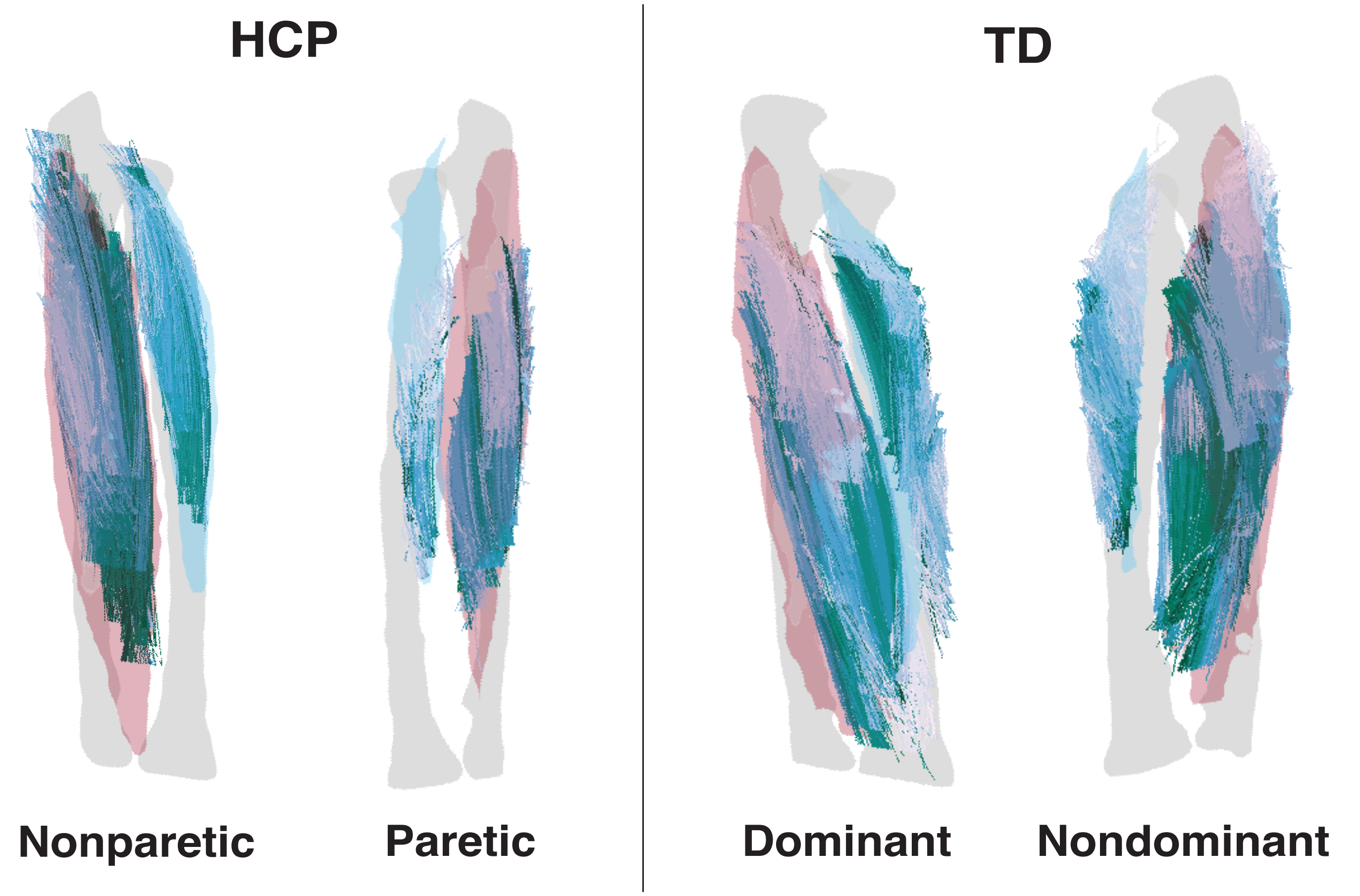

Figure S1. Examples of muscle fibers in forearm flexor muscles reconstructed from DTI-based probabilistic tractography. Fibers are shown overlaid on the flexor carpi radialis (FCR) muscle (blue) and the flexor digitorum profundus (FDP) muscle (red). The ulna and radius bones are shown in light grey.

Quantification of Intramuscular Fat in Two Individuals with HCP and Two TD Individuals

To ensure that changes in intramuscular fat did not affect the diffusivity metrics observed in this study, we performed a post-hoc analysis of intramuscular fat fraction in the forearm flexor muscles of both arms of two participants with HCP (26.42-year-old female, BMI=35.2; 20.17-year-old female, BMI=18.0) and two TD participants (26.5-year-old female, BMI=24.4; 24.8-year-old female, BMI=21.0).

Image acquisition: On a 1.5T scanner, 3D multi-echo mDixon scans were acquired with a Fast Field Echo sequence (TR=6ms, TE1=3.5ms, TE2=4.6ms, FOV=180x180 mm^2, 0.94x0.94x2 mm^3, averages=2). Arm, wrist, and hand position was kept the same as in the current study (Figure 1A).

Fat fraction analysis: Fat only and water only images were created by taking the difference, for fat only, or summing, for water only, of the in-phase and opposed-phase images from the mDixon scans. The mean and standard deviation of the fat signal intensity and water signal intensity across the segmented muscle region (FCR, FDP, or FCR and FDP together) was calculated (see below for schematic). Fat fraction was calculated according to equation (1) below[1].

Calculation of Fat Fraction

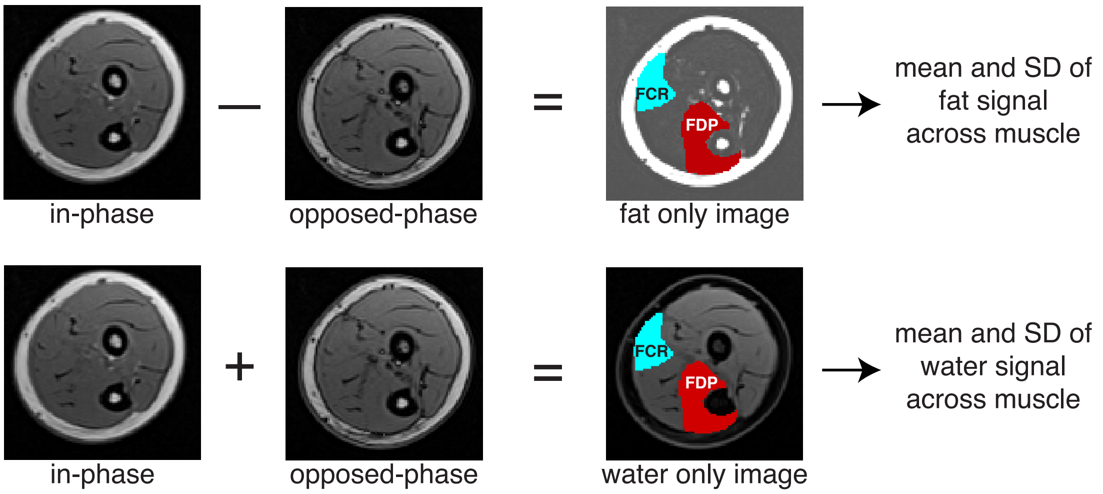

$$\boldsymbol{Fat\ Fraction}\ (\%) = \frac{\boldsymbol{\mu_{fat}}}{\boldsymbol{\mu_{fat}} + \boldsymbol{\mu_{water}}} * \mathbf{100\%} \qquad (1)$$

μ_{fat}: $mean\ signal\ intensity\ from\ fat\ only\ image$

μ_{water}: $mean\ signal\ intensity\ from\ water\ only\ image$

Results: Fat fraction values of forearm muscles are shown in Supplementary Figure 2. To determine the effect size between arms, Cohen's *d* was calculated between the arms for each muscle group and each participant. In all cases, *d* was less than 0.2, indicating that the difference in fat fraction between arms is negligible[2].

Conclusion: Given that the interlimb difference is negligible in all four participants, we can assume that there is no meaningful change in fat fraction in muscles affected by HCP or TD muscles, indicating that intramuscular fat fraction did not contribute to observed interlimb differences in diffusivity metrics. Furthermore, Williams et al. concluded that if the intramuscular fat fraction is less than 45%, diffusivity metric estimates are unaffected by fat content[3]. The fat fraction values observed here fall between 3-5%, much lower than the threshold for accurate diffusivity metric measurement. Finally, SPectral Attenuated Inversion Recovery (SPAIR) sequence was used to suppress any contributions from fat to the diffusion signal. Therefore, we believe that intramuscular fat infiltration did not significantly affect changes in diffusivity metrics observed in the current study.

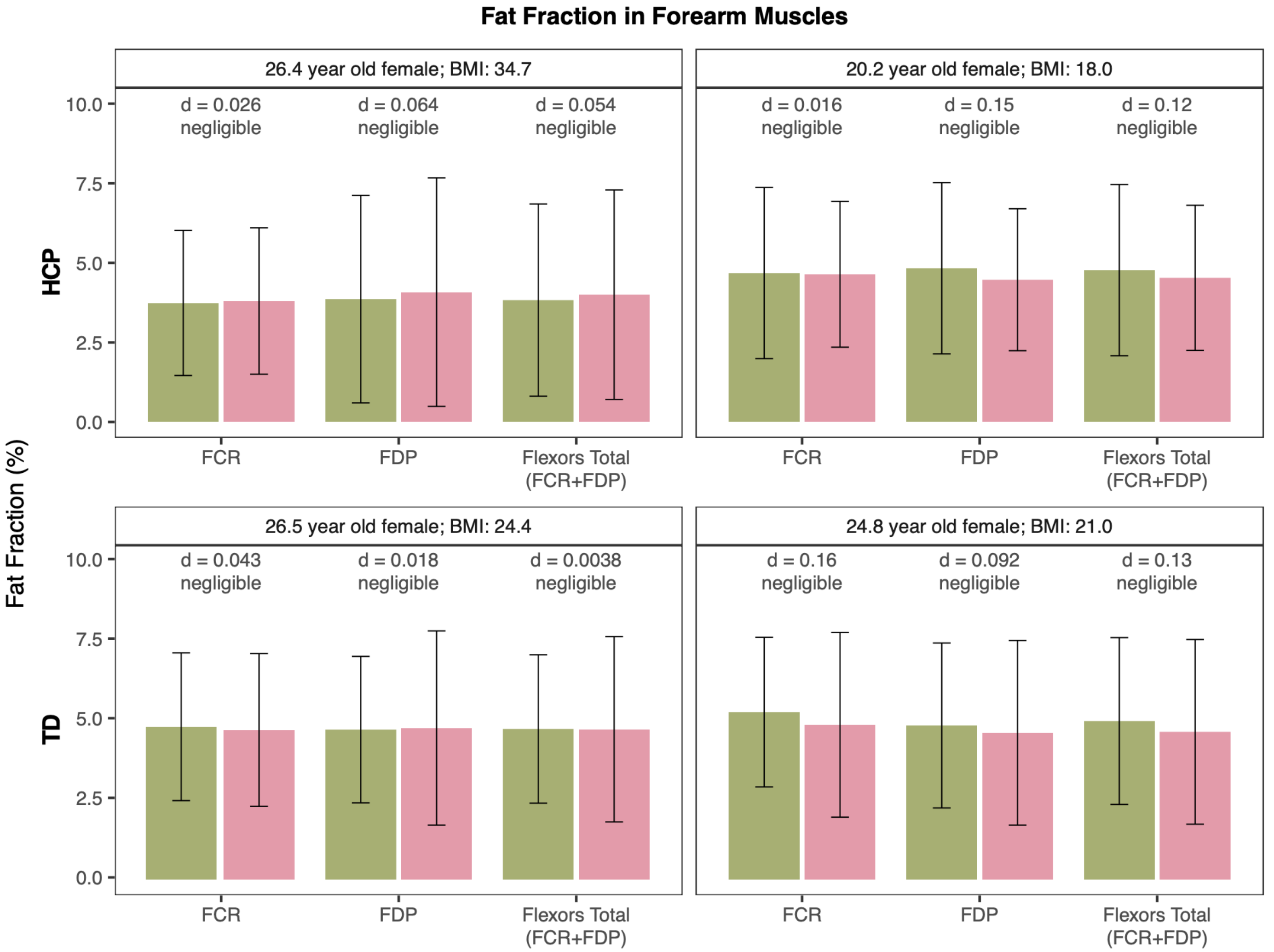

Figure S2. Fat fraction (%) in two participants with HCP (top row panels) and two TD participants (bottom row panels). Mean (colored bars, value in white at the bottom of each bar) and standard deviation (black error bars) values are shown for each arm (green: dominant/nonparetic, pink: nondominant/paretic) for the FCR, the FDP, and the FCR and FDP muscles summed together. Cohen's d values comparing fat fraction between arms in each arm and muscle group are shown at the top. The interlimb difference in each case is negligible (d<0.2)[2].

References

1 Dixon WT. Simple proton spectroscopic imaging. *Radiology* 1984;**153**:189–94. https://doi.org/10.1148/RADIOLOGY.153.1.6089263.

2 Cohen J. Statistical Power Analysis for the Behavioral Sciences. *Stat Power Anal Behav Sci* 2013. https://doi.org/10.4324/9780203771587.

3 Williams SE, Heemskerk AM, Welch EB, Li K, Damon BM, Park JH. Quantitative effects of inclusion of fat on muscle diffusion tensor MRI measurements. *J Magn Reson Imaging* 2013;**38**:1292–7. https://doi.org/10.1002/JMRI.24045.

Absolute Maximum Grip Strength versus Physiological Cross-Sectional Area

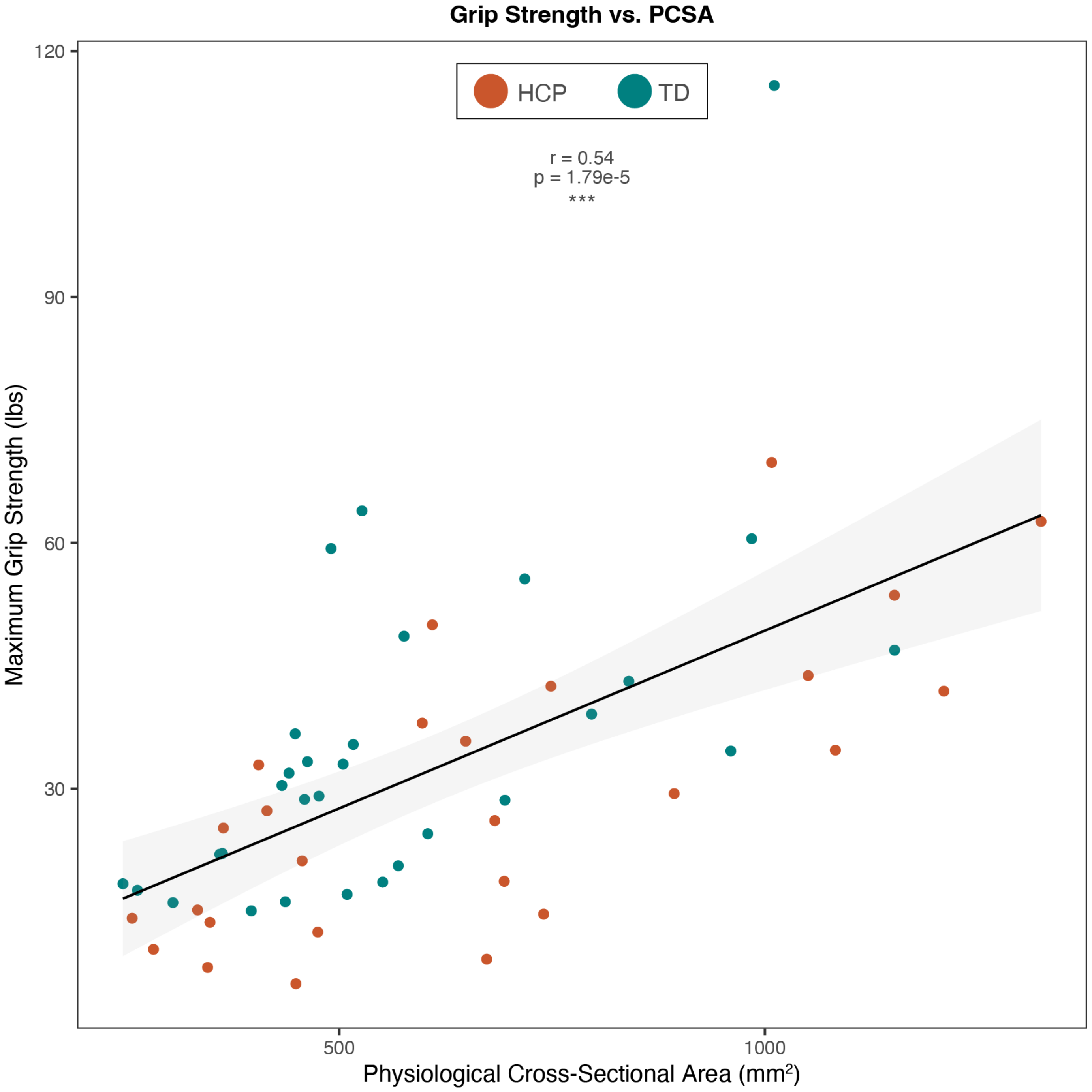

Figure S3. Maximum grip strength (lbs) versus physiological cross-sectional area (PCSA) (mm^2) across all participants and both arms. Red dots represent individuals with HCP and green dots represent TD individuals. Linear regression line fitted to the data is shown in black, with 95% confidence interval shown in light grey. Pearson correlation coefficient (r) and associated p-value is shown. *A *** indicates p<0.001.*
